# Awareness of audiology and speech-language pathology services among healthcare professionals in Saudi Arabia

**DOI:** 10.4102/sajcd.v71i1.1043

**Published:** 2024-06-07

**Authors:** Ahmad A. Alanazi, Mohammed F. ALHarbi, Abrar M. AlMutairi, Maryam A. AlRashied, Reham Abed

**Affiliations:** 1 Department of Audiology and Speech Pathology, College of Applied Medical Sciences, King Saud bin Abdulaziz University for Health Sciences, Riyadh, Saudi Arabia; 2 King Abdullah International Medical Research Center, Riyadh, Saudi Arabia; 3 Department of Speech-Language Pathology and Audiology, College of Medical Rehabilitation Sciences, Taibah University, Al-Madinah Al-Munawarrah, Saudi Arabia; 4 Research Unit, College of Applied Medical Sciences, King Saud bin Abdulaziz University for Health Sciences, Riyadh, Saudi Arabia; 5 Medical and Surgical Ward, King Saud Medical City, King Saud University, Riyadh, Saudi Arabia

**Keywords:** audiology, awareness, interprofessional practice, knowledge, Saudi Arabia, speech-language pathology

## Abstract

**Background:**

Healthcare professionals are required to work effectively together to deliver the best healthcare services. Without awareness of other healthcare professionals’ roles and responsibilities, interprofessional practice (IPP) cannot be optimally achieved.

**Objectives:**

This study aimed to investigate healthcare professionals’ awareness of audiology and speech-language pathology (SLP) services in Saudi Arabia.

**Method:**

This cross-sectional descriptive study consisted of two parts. The content of a 20-item paper questionnaire was firstly validated. The full-scale study addressed the aim through distributing questionnaire items among potential participants. Descriptive statistics and chi-square test were used.

**Results:**

A total of 403 participants completed the questionnaires for the main study. Most of the participants were Saudi citizens (84.1%), aged 18 years – 40 years (84.8%) years, and lived in Riyadh region (76.2%). Allied health professionals (40.2%), physicians (22.6%), nursing (15.4%) and dentistry (11.2%) were the main group of participants working mainly at governmental hospitals (69.2%). Of the total participants, 92.6% and 95.3% reported being fully aware of the services provided by audiologists and SLPs, respectively. No statistically significant association between the specialty of participants and their familiarity with the scope of practice for SLPs and audiologists was determined.

**Conclusion:**

Our study examined healthcare professionals’ awareness of audiology and SLP services and revealed a high level of awareness.

**Contribution:**

The existed level of awareness is expected to facilitate IPP and enhance the quality of care. Still, awareness campaigns about audiology and SLP services are needed to address the existing lack of knowledge among some healthcare professionals.

## Introduction

Audiologists have expertise in preventing, diagnosing and treating hearing and balance disorders among people of all ages, while speech-language pathologists (SLPs) have expertise in evaluating, diagnosing and treating speech, language and swallowing disorders across the human life span (American Speech-Hearing-Language Association [ASHA], 2016, 2018). Although audiology and speech-language pathology (SLP) are separate professions, both professions are usually linked with each other to identify, diagnose and intervene with individuals who have hearing and communication problems (ASHA, 2016, 2018). For example, audiologists determine a patient’s eligibility for a cochlear implant (CI), check the functioning and placement of electrodes during surgery and are responsible for mapping the CI and supporting with adjustment and ‘turning on’ of the speech processor after surgery (Prelock, 2013). Speech-language pathologists work closely with their colleagues in audiology to offer rehabilitation treatments, which encompass speech reading and auditory training (Prelock, 2013). Both guarantee improved communication and assist in meeting the needs of the patient and their family.

Audiology and SLP are considered new professions in Saudi Arabia (Alanazi, 2017). According to the General Authority for Statistics in Saudi Arabia, 1.4% and 1.1% of the total Saudi population suffer from mild, severe or extreme hearing and communication difficulties, respectively (General Authority for Statistics, 2017). Audiology and SLP services have been mainly accessed through referrals from other healthcare professionals, such as paediatricians, ear, nose, and throat (ENT) specialists and general practitioners or via direct access for non-acute conditions (British Academy of Audiology, 2021; Centers for Medicare and Medicaid Services, 2022; Molini-Avejonas et al., 2015). By referring patients directly to the appropriate healthcare provider, errors in diagnosis and treatment can be eliminated, and collaboration and coordination among healthcare professionals are enhanced (Blank et al., 2003; McNair, 2005).

Collaboration among healthcare professionals has emerged as a key component of improved health services. Interprofessional education (IPE) and interprofessional practice (IPP) foster the development of pertinent competencies in interprofessional communication, conflict resolution, leadership, patient-centred care and ethical practice (Setiadi et al., 2017; Yusra et al., 2019). Effective collaboration between healthcare professionals may reduce healthcare costs, increase patients’ safety and improve the quality of care (McNair, 2005; Setiadi et al., 2017). In contrast, separate-serving professionalism methods may create obstacles between health professions that affect the development of healthcare generally (McNair, 2005). For example, treating patients using uniprofessional approaches increases the cost (Learning and Teaching for Interprofessional Practice Australia, 2009). Therefore, the World Health Organization (WHO, 2010) reported that ‘In the current global climate, health workers also need to be interprofessional’.

Awareness of other healthcare professionals’ specialties and their scope of practice facilitate collaboration between healthcare professionals, and without such awareness, the core competencies for IPP, which are values and ethics, roles and responsibilities, interprofessional communication and teams and teamwork, cannot be optimally achieved. Consequently, the quality of care may be affected (Interprofessional Education Collaborative Expert Panel, 2011). The literature has frequently assessed the public awareness of audiology and SLP services in different populations (Alanazi & Al Fraih, 2021; Al Rjoob et al., 2022; Alshehri et al., 2019; Chu et al., 2019; Janes et al., 2021; Joubert et al., 2021) and ignored the awareness of both services among healthcare professionals. With the increased demand for audiology and SLP services in Saudi Arabia (Alanazi, 2017), healthcare professionals’ awareness of audiology and SLP should be investigated to facilitate IPP. Therefore, this study aimed to examine healthcare professionals’ awareness of audiology and SLP services in Saudi Arabia.

## Research methods and design

This cross-sectional descriptive study was designed to determine the awareness of audiology and SLP services among healthcare professionals in Saudi Arabia. No identifiable or health information was collected. Only the authors had access to the data from the completed questionnaires. The study included all healthcare professionals, who worked in Saudi Arabia at the time of the study and were not necessarily from Saudi originally, except audiologists and SLPs.

### The questionnaire

The questionnaire was developed by the first two authors (A.A.A. and M.F.A.) (Appendix 1). All the questions were developed in English, so there was no need for forward and background translation to Arabic because English is the professional language of healthcare professionals. The questionnaire consisted of 20 items distributed into three categories: demographic information, experience with hearing and communication disorders, and awareness of audiology and SLP services.

In the first category of the questionnaire, participants were asked 11 questions about their age, gender, nationality, region of work, health specialty, specialty rank (e.g. specialists, consultants, etc.), place of work, years of experience, marital status, whether they have children and the number of children if applicable. The second category included two questions about the participants’ experience of working with or referring patients to an audiologist or an SLP and whether they or anyone they know has ever been diagnosed with a hearing loss or communication disorder. The third category consisted of seven questions that examined the participants’ awareness of audiology and SLP services, which included where can audiologists and SLPs work, with whom audiologists and SLPs work, how much study and training would you expect an audiologist or SLP to have beyond graduating high school, whether an SLP and a special education teacher do the same work, whether or an audiologist and a deaf and hard-of-hearing teacher do the same work, what is the job of an SLP and what is the job of an audiologist.

#### The pilot questionnaire

Hard copies of the questionnaire were distributed to 20 participants from different healthcare professions for piloting. The participants were required to read and fill out an informed consent form prior to filling out and submitting the questionnaire. The sample size of the main study did not include the 20 pilot questionnaire participants. The participants were asked questions regarding the clarity of each question to make sure there was no misinterpretation. They reported that the questions were clear. The final version of the questionnaire was found to have both internal consistency and face validity. Statistical Package for Social Sciences (SPSS) for Windows v. 26.0 (IBM SPSS Statistics, IBM Corporation, Armonk, NY, USA) was used to analyse the data of 20 participants. The questionnaire was validated using principal components analysis (PCA) and Cronbach’s α score calculations. The results of the KMO and Bartlett’s test showed that the variables are significantly correlated on PCA and a Cronbach’s α score of 0.78 was attained. No changes were made to the questionnaire.

### The main study

In this study, a convenience sampling technique was employed. The electronic version of the questionnaire was created using Google Forms (Google LLC, Mountain View, California, SA) to reach many people quickly and affordably throughout Saudi Arabia. A random sample of healthcare professionals received the questionnaire link via email sent by their employers. Moreover, the link to the questionnaire was distributed and shared via social media (X [formerly Twitter], LinkedIn and WhatsApp). The questionnaire link was accessible for 8 months with monthly reminders. Furthermore, some of the responses were collected by filling out hard copies of the questionnaire through face-to-face separate meetings conducted by three authors (A.A.A., A.M.A. and R.A.) with the potential participants at different healthcare settings. The total number of healthcare professionals in Saudi Arabia is around 423 940 (Ministry of Health, 2017), and the sample size was calculated to be 384 participants using Raosoft sample size calculator (Raosoft, Inc., USA) with 5% margin of error, 95% confidence level and 50% response distribution. Participation was optional and answers were anonymous. The collected sample size included 403 participants.

Statistical analysis was carried out using SPSS for Windows v. 26.0 software. Descriptive statistics were used to present socio-demographic data for categorical variables, which included frequency and percentages. Chi-square test was used to find the association between the level of awareness of both services and (1) whether participants were diagnosed or ever known an individual diagnosed with a hearing loss or communication disorder, (2) participants’ specialty and (3) whether participants have children. The above independent variables were examined because they may positively affect the level of awareness of audiology and SLP services among healthcare professionals (i.e. the dependent variable) not because of the participants’ daily work in healthcare settings. Furthermore, univariate regression analysis was conducted to analyse the association between knowledge of the scope of practice of SLPs and audiologists and whether the participants have children. This association was identified based on the odds ratio (OR) and 95% confidence interval (CI). A *p*-value less than 0.05 was considered statistically significant.

### Ethical considerations

Ethical approval to conduct this study was obtained from the King Abdullah International Medical Research Centre (KAIMRC) Institutional Review Board.

## Results

The study included 403 (male: *n* = 144; female: *n* = 259) participants with nearly half (47.6%) aged between 18 years and 30 years (Table 1). Most of the participants were Saudi, comprising 84.1% of the total. Most of the participants were from Riyadh region (67.2%), followed by Madinah (13.9%) and Makkah (6.2%) regions, respectively. Regarding specialty, most of the participants were from different allied health professions (e.g. physical therapy, clinical nutrition, medical laboratory and occupational therapy) accounting for 40.2%, followed by 22.6% from medicine, 15.6% from nursing and 11.2% from dentistry (Table 1). In terms of employment, most of the participants included in this study worked at governmental hospitals (69.2%), followed by universities (18.4%) and private hospitals (10.7%). Among the participants, 32% were specialists, and the remaining were 12.4% consultants, 15.1% interns, 14.6% technologists and 12.8% residents. Additionally, 45.7% of the participants had an experience ranging from 1 to 5 years.

**TABLE 1:** Demographic data of included participants.

Variables	*n*	%
**Gender**		
Male	144	35.7
Female	259	64.3
**Age (years)**		
18–30	192	47.6
31–40	150	37.2
41–50	45	11.2
51–60	11	2.7
61–70	5	1.0
**Nationality**		
Saudi	339	84.1
Non-Saudi	64	15.9
**Region**		
Riyadh	271	67.2
Makkah	25	6.2
Madinah	56	13.9
Qassim	12	3.0
Jawf	4	1.0
Eastern Province	11	2.7
Ha’il	6	1.5
Jizan	2	0.5
Asir	16	4.0
**Specialty**		
Allied health professions	162	40.2
Medicine	91	22.6
Dentistry	45	11.2
Pharmacy	13	3.2
Nursing	63	15.6
Other (e.g. biomedical engineering, patient care technician)	29	7.2
**Work area**		
Governmental hospital	279	69.2
Research centre	5	1.2
University	74	18.4
Private hospital	43	10.7
Unemployed	2	0.5
**Current credential**		
Consultant	50	12.4
Fellow	5	1.2
Senior specialist	31	7.7
Specialist	129	32.0
Intern	61	15.1
Resident	49	12.8
Registrar	17	4.2
Technologist	59	14.6
**Experience (years)**		
1–5	184	45.7
11–15	50	12.4
6–10	78	19.4
16 or more	40	9.9
No experience	51	12.7
**Marital status**		
Single	216	53.6
Married	177	43.9
Divorced	10	2.5
**Children**		
Yes	162	40.2
No	241	59.8
**Number of children**		
2 or less	93	22.8
3–5	64	15.9
≥ 6	5	1.2

More than half of the participants (64%) reported that they had not worked with or referred any patients to audiologists or SLPs. Only 14.9% of the participants reported they worked with both audiologists and SLPs (Table 2). Regarding the work settings, participants were allowed to select more than one option. More than half (85.7%) of the respondents indicated that audiologists and SLPs work in hospitals, while 63.1% mentioned private clinics and 62.1% rehabilitation centres as the workplace of audiologists and SLPs. Regarding what age group that audiologists and SLPs work with, participants were allowed to select more than one option. Of the total participants, 71.4% of them reported that audiologists and SLPs work only with school-aged children and 70.4% work with toddlers and preschoolers. For the study and training duration for audiologists or SLPs beyond graduating high school, only 29.3% of the respondents reported around 5 years (Table 2). In addition, 80.4% of the respondents indicated that SLPs and special education teachers do not have the same job, while 81.6% reported that audiologists and deaf and hard-of-hearing teachers do not have the same job. When the participants were asked about their knowledge of the job of audiologists and SLPs, their answers were divided into two categories: true category (i.e. correctly identified the job) and false category (i.e. incorrectly identified the job). The majority of the participants (95.3%) reported being fully aware of the job of SLPs and 92.6% stated being aware of the job of audiologists (Table 2).

**TABLE 2:** Experience with hearing and communication disorders and awareness of audiology and speech-language pathology.

Survey items	*n*	%
**Have you ever worked with or referred patients to an audiologist or speech-language pathologist?†**		
Both	60	14.9
Audiologist	40	9.9
Speech-language pathologist	45	11.2
No	258	64.0
**During your lifetime, have you or anyone you know ever been diagnosed with a hearing loss or communication disorder?†**		
Both	50	12.4
Communication disorder	26	6.5
Hearing loss	98	24.3
Autism	4	1.0
Other (e.g. ADHD)	6	1.5
No	218	54.1
**Where can audiologists and speech-language pathologists work?†**		
Hospitals	258	85.7
Kindergartens	111	36.9
Universities	121	40.2
Schools	150	49.8
Private clinics	190	63.1
Military	59	19.6
Nursing homes	97	32.3
Rehabilitation centres	187	62.1
**Who do audiologists and speech-language pathologists work with?†**		
Infants	110	36.5
Toddlers and preschool	212	70.4
School-aged children	215	71.4
Adolescent	165	54.8
Adults	195	64.8
Elderly	161	53.5
Do not know	24	0.8
**How much study and training would you expect an audiologist or speech-language pathologist to have beyond graduating high school?**		
At least 1 year	62	15.4
2–3 years	87	21.6
4 years	99	24.6
5 years	118	29.3
> 5 years	37	9.2
**A speech-language pathologist and a special education teacher do the same work**		
Yes	79	19.6
No	324	80.4
**An audiologist and a deaf and hard-of-hearing teacher do the same work**		
Yes	74	18.4
No	329	81.6
**What is the job of a speech-language pathologist?†**		
True	384	95.3
False	19	4.7
**What is the job of an audiologist?** ‡		
True	373	92.6
False	30	7.4

† , Participants were allowed to select more than one option;

‡ , True: correctly identified the job; False: incorrectly identified the job.

More than half of the participants (*n* = 218) reported not being diagnosed with hearing loss or communication disorders with only 24.3% and 12.4% reported yes for hearing loss and both hearing loss and communication disorders, respectively (Table 2). There was no statistically significant association between the participants’ awareness of the job of audiologists and whether the participants have been diagnosed or have ever known an individual diagnosed with a hearing loss, as indicated by a *p*-value of 0.41. Similarly, no statistically significant association (*p*-value = 0.52) was found between the participants’ awareness of the job of audiologists and their specialty (Table 1-A2, Appendix 2). Moreover, no statistically significant association between the participants’ awareness of the job of SLPs and whether the participants have been diagnosed or ever know individuals diagnosed with a communication disorder, as indicated by a *p*-value of 0.92. Also, no statistically significant association (*p*-value = 0.50) was found between the participants’ awareness of the job of SLPs and their specialty (Table 1-A3, Appendix 3). The regression analysis examining the association between knowledge about the job of audiologists and SLPs with whether the participants have children indicated no statistical significance (Table 3); the *p*-values were 0.11 and 0.68, respectively.

**TABLE 3:** Regression analysis for the association between knowledge about the job of audiologists and speech-language pathologists with whether the participants have children.

Participants’ knowledge	Odds ratio	CI 95%	*p*
**Audiologists’ job**			
Yes	0.47	0.18–1.19	0.11
No	Ref	Ref	Ref
**SLPs’ job**			
Yes	1.17	0.54–2.53	0.68
No	Ref	Ref	Ref

CI 95%, confidence interval at 95%; Ref, reference; SLPs, speech-language pathologists.

## Discussion

The career of audiology and SLP includes working with a wider range of individuals with different stages of disabilities and diseases, including acute, subacute and chronic stages in varied settings. Both audiologists and SLPs work with a diverse group of healthcare professionals, who are expected to be familiar with the scope of practice of both specialists. Successful collaboration among audiologists, SLPs and other healthcare professionals to address hearing, vestibular, communication and swallowing disorders leads to the provision of comprehensive services, achieving better outcomes and higher satisfaction with time and cost efficiency (Welling & Ukstins, 2023). Investigating the awareness of healthcare professionals about audiology and SLP as team members is one way to promote IPP. This study measured the awareness of audiology and SLP services among healthcare professionals in Saudi Arabia. A total of 403 healthcare professionals participated in the main study, which is a good response rate above the minimum effective sample size. Our results showed an overall high level of awareness of audiology and SLP services.

Most of the participants were young Saudi female participants (*n* = 259 [64.3%]; aged 18 years – 40 years [84.8%]) from three main regions, including Riyadh, Madinah and Makkah, respectively. Audiology and SLP services are well established in different healthcare settings in three large regions Riyadh, Eastern and Makkah regions (Alanazi & Al Fraih, 2022; Khoja & Sheeshah, 2018). For example, Riyadh has 102 hospitals with more than 18 780 beds (Ministry of Health, 2017). Allied health professionals working in governmental and private hospitals and universities were the majority of participants. Only a few consultants participated in this study, thus the working experience was limited to less than 5 years. Allied health professionals who are credentialed as specialists (75%) are the major health workforce in Saudi Arabia (Ministry of Health, 2017). Only a few participants reported working with or referring patients to both audiologists and SLPs even though they exhibited a high level of awareness. This could occur because of the referral and access to the services system. Most of the participants worked at hospitals where audiology and SLP services are mainly accessed after the ENT referral pathway, so limited contact with audiologists and SLPs is expected. Furthermore, the lack of such services in other cities may lead to such limited collaboration. Alanazi investigated audiology and SLP practice in Saudi Arabia, and he reported that most of the participants in his study were from Riyadh, which may explain the lack of services in other Saudi cities (Alanazi, 2017). This challenge of lack of services and limited collaboration between audiologists and SLPs and with healthcare professionals can be addressed through IPE and IPP and effectively utilising tele-practice to promote audiology and SLP services in regions where such services are either limited or do not exist.

More than half (85.7%) of the respondents indicated that both audiologists and SLPs work in hospitals, followed by private clinics and rehabilitation centres. Our results demonstrated a good understanding of potential workplaces for these professionals, despite the paucity of information regarding these professionals’ employment and present practices in Saudi Arabia (Alanazi, 2017). The majority of participants accurately concluded that the most common places of employment for audiologists and SLPs were hospitals, private clinics and rehabilitation facilities. According to estimates, hospitals employ the majority of Saudi Arabia’s audiologists and SLPs (Alanazi, 2017). There are not many audiologists and SLPs working in Saudi schools, regardless of the proportion of participants who chose this setting as a possible employment opportunity (Alanazi, 2017; Alquraini, 2010). Many in the education sector firmly believe that services provided by both audiologists and SLPs in schools are necessary (AlAbdulkarim, 2015).

Furthermore, most of the participants chose that audiologists and SLPs work only with school-aged children followed by toddlers and preschoolers. This could be because of limited knowledge of the disorders that affect hearing and communication in adults or a limited number of rehabilitation centres across the country where adults are being seen or treated for neurological disorders affecting communication, such as stroke (Alanazi & Al Fraih, 2021). Moreover, although audiology and SLP academic programmes are increasing in Saudi Arabia, the number of current programmes is limited with a few graduates, which could be a contributing factor to the shortage of the participants’ knowledge about where both audiologists and SLPs work and which age groups they serve. Speech-language pathologists and audiologists deal with people of various ages in Saudi Arabia, from newborns to adults (Alanazi, 2017). For the question about study and training duration for audiologists or SLPs beyond graduating high school, only 29.3% of the respondents reported around 5 years followed by 4 years and 2 to 3 years, respectively. The minimum educational need to become an audiologist or SLP in Saudi Arabia is a bachelor’s degree that consists of 4 years of education, plus one training year (i.e. internship) (AlAkeel, 2022; Alanazi, 2017). Most participants distinguished between audiologists and teachers of the deaf and hard of hearing, as well as between SLPs and special education instructors. Despite being partners in education, audiologists, SLPs, teachers of the deaf and hard of hearing, and special education instructors have distinct areas of expertise. Yet, our result showed that approximately 20% of the participants still neither differentiate between the role of SLPs and the role of special education teachers nor between the role of audiologists and the role of deaf and hard-of-hearing teachers, which indicates the need for increasing awareness about audiologists and SLPs scope of practice.

The majority of the participants (95.3%) reported being fully aware of the job of SLPs and 92.6% stated being aware of the job of audiologists. The participants were aware that performing surgery or prescribing medicine is under the scope of practice of audiologists and SLPs. However, some of the audiology services, such as tinnitus and vestibular assessment and rehabilitation or SLP services, such as voice and swallowing disorders diagnosis and rehabilitation are not regularly practised in all healthcare settings in Saudi Arabia because of the lack of training and equipment (AlAkeel, 2022; Alanazi, 2017). In our study, most of the participants (40.2%) were from allied health professions, who correctly identified the scope of practice of both audiologists and SLPs. This could be because allied health professionals usually graduate from colleges of applied medical sciences in Saudi universities, which encompass audiology and SLP professions. No statistically significant association between knowledge about the scope of practice of audiologists and SLPs with whether the participants have children was identified. This finding contrasts with a study conducted by Mahmoud et al. (2014), which showed that the majority of female participants who have children and hold a bachelor’s degree in health or education fields possessed knowledge about SLP and communication disorders. Having a child with hearing loss or a communication disorder requires parents to investigate the healthcare services their child needs, which may positively affect their awareness of audiology or SLP services. The rehabilitation of children with hearing loss and/or a communication disorder requires collaborative practice between audiologists and/or SLPs and parents (Klatte et al., 2020; Meibos et al., 2016).

### Strengths and limitations

To the best of our knowledge, this is the first study that investigated the awareness of audiology and SLP services among healthcare professionals in Saudi Arabia. Although the questionnaire used in this study was developed and validated to serve the aim of the study, it is a new questionnaire that was not validated in other countries. The correlation of variables, such as current credentials and experience with the level of awareness of both audiology and SLP services was not investigated. Despite having a sufficient sample size from different healthcare specialties, the sample did not adequately represent all Saudi regions.

### Future research and recommendations

Research is needed to find out the level of awareness about specific hearing and communication impairments among healthcare professionals in Saudi Arabia. Interprofessional practice among audiologists, SLPs and other healthcare professionals in Saudi Arabia needs to be investigated. It is recommended that academic institutions include IPE and IPP in their curricula to enhance understanding of what other disciplines can provide in terms of roles and responsibilities (Baker et al, 2005):

Teamwork requires a shared acknowledgement of each participating member’s roles and abilities. Without this acknowledgement, adverse outcomes may arise from a series of seemingly trivial errors that effective teamwork could have prevented. (p. 14)

## Conclusion

Audiologists and SLPs provide a variety of services under their scope of practice in many Saudi healthcare facilities. With the increased demand for audiology and SLP services in Saudi Arabia, this study examined healthcare professionals’ awareness of audiology and SLP services. The findings indicate an overall high level of awareness existing among healthcare professionals. Still, our result showed that some of the participants neither differentiate between the role of SLPs and the role of special education teachers nor between the role of audiologists and the role of deaf and hard-of-hearing teachers. It is the responsibility of SLPs and audiologists to raise awareness, particularly among healthcare professionals who still lack or have limited awareness of both specialties, and educate their colleagues about the services they provide as part of interprofessional education. Future research should focus on IPP among audiologists and/or SLPs with other healthcare professionals in Saudi Arabia.

## Appendix 1

### Questionnaire

#### Section 1: Demographic information

1. What is your gender?
  - Male
  - Female
2. What is your age (years)?
  - 18–30
  - 31–40
  - 41–50
  - 51–60
  - 61–70
  - 71 and over
3. What is your nationality?
  - Saudi
  - Non-Saudi
4. In which region do you live?
  - Riyadh
  - Makkah
  - Madinah
  - Qassim
  - Tabuk
  - Jawf
  - Northern Borders
  - Eastern Province
  - Ha’il
  - Baha
  - Jizan
  - Asir
  - Najran
5. What is your specialty?
  - Medicine (please specify)
  - Allied health professions (please specify)
  - Dentistry
  - Nursing
  - Pharmacy
  - Other (please specify)
6. Where do you work?
  - Governmental hospital
  - Private hospital
  - Nursing home
  - Rehabilitation centre
  - University
  - Military
  - Other (please specify)
7. What is your current credentials?
  - Consultant
  - Senior registrar
  - Registrar
  - Senior specialist
  - Specialist
  - Resident
  - Fellow
  - Technologist
  - Intern not credentials
8. How many years of experience do you have?
  - 1–5
  - 6–10
  - 11–15
  - 16 and more
  - No professional experience
9. What is your marital status?
  - Single
  - Married
  - Divorced
  - Widowed
10. Do you have children?
  - Yes
  - No
11. If yes, please indicate how many children you have:
  - 2 children or fewer
  - 3–5 children
  - 6 or more children

#### Section 2: Experience with hearing and communication disorders

12. Have you ever worked with or referred patients to an audiologist or a speech-language pathologist?
  - Yes, audiologist only
  - Yes, speech-language pathologist only
  - Yes, both
  - No
13. During your lifetime, have you ever been diagnosed with a hearing loss or communication disorder?
  - Yes, hearing loss
  - Yes, communication disorder (Please indicate type in the space provided below)
  - Yes, both hearing loss and communication disorder
  - No

#### Section 3: Awareness of audiology and SLP

14. Where can audiologists and speech-language pathologists work? (You may select one or more)
  - Kindergartens
  - Schools
  - Universities
  - Hospitals
  - Private clinics
  - Military
  - Nursing homes
  - Rehabilitation centres
  - Other (please specify)
15. Whom do audiologists and speech-language pathologists work with? (You may select one or more)
  - Infants
  - Toddlers and preschoolers
  - School-aged children
  - Adolescents
  - Adults
  - Elderly people
  - Not sure
16. How much study and training would you expect an audiologist or speech-language pathologist to have beyond graduating high school?
  - At least 1 year
  - 2–3 years
  - 4 years
  - 5 years
  - More than 5 years
17. An audiologist and a teacher of deaf and hard of hearing do the same work.
  - Yes
  - No
18. A speech-language pathologist and a special education teacher do the same work.
  - Yes
  - No
19. What is the job of an audiologist? (You may select one or more)
  - Diagnose hearing loss
  - Improve a person’s hearing
  - Prescribe and fit hearing aids and assistive listening devices
  - Diagnose and manage tinnitus
  - Diagnose and manage vestibular disorders
  - Prescribe medications
  - Provide surgical intervention
  - Other (please specify)
20. What is the job of a speech-language pathologist? (You may select one or more)
  - Diagnose and manage communication disorders (e.g. aphasia, apraxia, and stuttering)
  - Improve a person’s speech
  - Improve a person’s understanding of language
  - Diagnose and manage voice disorders
  - Diagnose and manage swallowing disorders
  - Prescribe medications
  - Provide surgical intervention
  - Other (please specify)

## Appendix 2

**TABLE 1-A2:** Association between how correctly the participants identified the job of audiologists with whether themselves or anyone they know had ever been diagnosed with a hearing loss or communication disorder and specialty.

Survey items	True†		False†		*p* ‡
	*n*	%	*n*	%	
**Have you or anyone you know ever been diagnosed with hearing loss?**					0.41
Both	48	12.9	2	6.7	
Communication disorder	26	7.0	0	0	
Hearing loss	92	24.7	6	20.0	
Autism	4	1.1	0	0	
Other (e.g. ADHD)	6	1.6	0	0	
No	196	52.7	22	73.3	
**Specialty**					0.52
Allied health professions	152	40.9	10	36.7	
Medicine	86	23.1	5	15.7	
Dentistry	40	10.7	5	15.7	
Pharmacy	11	2.9	2	10.0	
Nursing	57	15.3	6	16.9	
Other	27	7.2	1	5.0	

ADHD, Attention-Deficit/ Hyperactivity Disorder.

† , True: correctly identified the job; False: incorrectly identified the job;

‡ , Pearson’s chi-square (χ^2^) test was used.

## Appendix 3

**TABLE 1-A3:** Association between how correctly the participants identified the job of speech-language pathologists with whether themselves or anyone they know had ever been diagnosed with a hearing loss or communication disorder or specialty.

Survey items	True†		False†		*p* ‡
	*n*	%	*n*	%	
**Have you or anyone you know ever been diagnosed with a communication disorder?**					0.92
Both	48	12.5	2	10.5	
Communication disorder	26	6.8	0	0	
Hearing loss	93	24.3	5	26.3	
Autism	4	1.0	0	0	
Other (e.g. ADHD)	6	1.6	0	0	
No	206	53.8	12	63.2	
**Specialty**					0.50
Allied health professions	157	40.9	5	30.6	
Medicine	87	22.7	4	20.1	
Dentistry	41	10.7	4	20.1	
Pharmacy	12	3.1	1	7.0	
Nursing	60	15.6	3	15.2	
Other	27	7.0	1	7.0	

ADHD, Attention-Deficit/ Hyperactivity Disorder.

† , True: correctly identified the job; False: incorrectly identified the job;

‡ , Pearson’s chi-square (χ^2^) test was used.

## References

[CIT0001] AlAbdulkarim, L. (2015). The role of speech-language pathologists and audiologists in the schools in Saudi Arabia. *International Journal of Health Economics and Management*, 1(2), 62–69.

[CIT0002] AlAkeel, A.I. (2022). The practice of speech-language pathology in Saudi Arabia. *Arab Journal of Applied Linguistics*, 7(1), 87–99. Retrieved from https://files.eric.ed.gov/fulltext/EJ1375033.pdf

[CIT0003] Alanazi, A.A. (2017). Audiology and speech-language pathology practice in Saudi Arabia. *International Journal of Health Sciences*, 11(5), 43–55. Retrieved from https://pubmed.ncbi.nlm.nih.gov/29114194/29114194 PMC5669511

[CIT0004] Alanazi, A.A., & Al Fraih, S.S. (2021). Public awareness of audiology and speech-language pathology in Saudi Arabia. *Majmaah Journal of Health Sciences*, 9(2), 36–51. 10.5455/mjhs.2021.02.005

[CIT0005] Alquraini, T. (2010). The Saudi education system. *International Journal of Science Education*, 25(3), 139–147. Retrieved January 17, 2024 from https://files.eric.ed.gov/fulltext/EJ909292.pdf

[CIT0006] Al Rjoob, M.A., Al Rjoob, K., & Kharraz, B.A. (2022). Public awareness of speech – Language pathology and audiology: A pilot study in Jordan. *Tunisie Medical*, 100(5), 384–389. Retrieved from https://www.ncbi.nlm.nih.gov/pmc/articles/PMC9552244/PMC9552244 36206087

[CIT0007] Alshehri, K.A., Alqulayti, W.M., Yaghmoor, B.E., & Alem, H. (2019). Public awareness of ear health and hearing loss in Jeddah, Saudi Arabia. *South African Journal of Communication Disorders*, 66(1), e1–e6. 10.4102/sajcd.v66i1.633 PMC6739561 31478749

[CIT0008] American Speech-Language-Hearing Association. (2016). *Scope of practice in speech language pathology*. Retrieved from www.asha.org/policy/

[CIT0009] American Speech-Language-Hearing Association. (2018). *Scope of practice in audiology*. Retrieved January 21, 2024 from www.asha.org/policy/

[CIT0010] Baker, D.P., Gustafson, S., Beaubien, J.M., Salas, E., & Barach, P. (2005). *Medical teamwork and patient safety: The evidence-based relation. Literature Review*. AHRQ Publication No. 05-0053. Agency for Healthcare Research and Quality. Retrieved from http://www.ahrq.gov/qual/medteam/

[CIT0011] Blank, L., Kimball, H., McDonald, W., Merino, J., ABIM Foundation, ACP Foundation, European Federation of Internal Medicine. (2003). Medical professionalism in the new millennium: A physician charter 15 months later. *Annals of Internal Medicine*, 138(10), 839–841. 10.7326/0003-4819-138-10-200305200-00012 12755556

[CIT0012] British Academy of Audiology. (2021). Guidance for audiologists: Onward referral of adults with hearing difficulty directly referred to audiology services. Retrieved January 10, 2024 from https://www.baaudiology.org/app/uploads/2019/07/BAA_Guidance_for_Onward_Referral_of_Adults_with_Hearing_Difficulty_Directly_Referred_to_Audiology_2016_-_minor_amendments.pdf

[CIT0013] Centers for Medicare and Medicaid Services. (2022). Audiology services. Retrieved January 15, 2024 from https://www.cms.gov/medicare/payment/fee-schedules/physician/audiology-services

[CIT0014] Chu, S.Y., Tang, K.P., McConnell, G., Rasdi, H.F.M., & Yuen, Mc. (2019). Public perspectives on communication disorders and profession of speech-language pathology. *Speech, Language and Hearing*, 22(3), 172–182. 10.1080/2050571X.2019.1570705

[CIT0015] General Authority for Statistics. (2017). Disability survey. Retrieved February 5, 2024 from https://www.stats.gov.sa/sites/default/files/disability_survey_2017_en.pdf

[CIT0016] Interprofessional Education Collaborative Expert Panel. (2011). *Core competencies for interprofessional collaborative practice: Report of an expert panel*. Interprofessional Education Collaborative. Retrieved February 8, 2024 from https://ipec.memberclicks.net/assets/2011-Original.pdf

[CIT0017] Janes, T.L., Zupan, B., & Signal, T. (2021). Community awareness of speech pathology: A regional perspective. *Australian Journal of Rural Health*, 29(1), 61–70. 10.1111/ajr.12680 33274537

[CIT0018] Joubert, K., Sebothoma, B., & Kgare, K.S. (2017). Public awareness of audiology, hearing and hearing health in the Limpopo Province, South Africa. *South African Journal of Communication Disorders*, 64(1), e1–e9. 10.4102/sajcd.v64i1.557 PMC5843015 29041791

[CIT0019] Klatte, I.S., Lyons, R., Davies, K., Harding, S., Marshall, J., McKean, C., & Roulstone, S. (2020). Collaboration between parents and SLTs produces optimal outcomes for children attending speech and language therapy: Gathering the evidence. *International Journal of Language & Communication Disorders*, 55(4), 618–628. 10.1111/1460-6984.12538 32383829 PMC7383473

[CIT0020] Khoja, M., & Sheeshah, H. (2018). The human right to communicate: A survey of available services in Saudi Arabia. *International Journal of Speech-Language Pathology*, 20(1), 102–107. 10.1080/17549507.2018.1428686 29400571

[CIT0021] Learning and Teaching for Interprofessional Practice Australia. (2009). *Interprofessional health education in Australia: The way forward*. Retrieved January 17, 2024 from https://ltr.edu.au/resources/LTIPP%20position%20paper%20v1.0.pdf

[CIT0022] Mahmoud, H.N., Al-Jazi, A.B., & Alkhamra, R.A. (2014). A study of public awareness of speech-language pathology in Amman. *College Student Journal*, 48, 495–510.

[CIT0023] McNair, R.P. (2005). The case for educating health care students in professionalism as the core content of interprofessional education. *Medical Education*, 39(5), 456–464. 10.1111/j.1365-2929.2005.02116.x 15842679

[CIT0024] Meibos, A., Muñoz, K., White, K., Preston, E., Pitt, C., & Twohig, M. (2016). Audiologist practices: Parent hearing aid education and support. *Journal of the American Academy of Audiology*, 27(4), 324–332. 10.3766/jaaa.15007 27115242

[CIT0025] Molini-Avejonas, D.R., Estevam, S.F., & Couto, M.I. (2015). Organization of the referral and counter-referral system in a speech-language pathology and audiology clinic-school. *CoDAS*, 27(3), 273–278. 10.1590/2317-1782/20152014158 26222945

[CIT0026] Ministry of Health. (2017). *Annual statistical book*. Retrieved January 21, 2024 from https://www.moh.gov.sa/en/Ministry/Statistics/book/Documents/ANNUAL-STATISTICAL-BOOK-1438H.pdf

[CIT0027] Prelock, P. (2013). Audiology and speech-language pathology: The magic of our connection. *ASHA Leader*, 18(12), 6–7. 10.1044/leader.FTP.18122013.6

[CIT0028] Setiadi, A.P., Wibowo, Y., Herawati, F., Irawati, S., Eko Setiawan, E., Presley, B. et al. (2017). Factors contributing to interprofessional collaboration in Indonesian health centres: A focus group study. *Journal of Interprofessional Education & Practice*, 8, 69–74. 10.1016/j.xjep.2017.06.002

[CIT0029] Welling, D.R., & Ukstins, C.A. (2023). Fundamentals of audiology for the speech-language pathologist. *Faculty Publications Books*. Retrieved February 20, 2024 from https://scholarship.shu.edu/faculty-pubs-books/4

[CIT0030] World Health Organization. (2010). *Framework for action on interprofessional education and collaborative practice*. Retrieved February 19, 2024 from https://www.who.int/publications/i/item/framework-for-action-on-interprofessional-education-collaborative-practice 21174039

[CIT0031] Yusra, R.T., Findyartini, A., & Soemantri, D. (2019). Healthcare professionals’ perceptions regarding interprofessional collaborative practice in Indonesia. *Journal of Interprofessional Education & Practice*, 15, 24–29. 10.1016/j.xjep.2019.01.005

